# Myostatin promotes distinct responses on protein metabolism of skeletal and cardiac muscle fibers of rodents

**DOI:** 10.1590/1414-431X20176733

**Published:** 2017-10-19

**Authors:** L.H. Manfredi, S. Paula-Gomes, N.M. Zanon, I.C. Kettelhut

**Affiliations:** 1 Departamento de Fisiologia, Faculdade de Medicina de Ribeirão Preto, Universidade de São Paulo, Ribeirão Preto, SP, Brasil; 2 Curso de Medicina, Universidade Federal de Fronteira Sul, Chapecó, SC, Brasil; 3 Departamento de Bioquímica e Imunologia, Faculdade de Medicina de Ribeirão Preto, Universidade de São Paulo, Ribeirão Preto, SP, Brasil

**Keywords:** Myostatin, Skeletal muscle, Cardiomyocytes, Protein degradation, Protein synthesis

## Abstract

Myostatin is a novel negative regulator of skeletal muscle mass. Myostatin expression is also found in heart in a much less extent, but it can be upregulated in pathological conditions, such as heart failure. Myostatin may be involved in inhibiting protein synthesis and/or increasing protein degradation in skeletal and cardiac muscles. Herein, we used cell cultures and isolated muscles from rats to determine protein degradation and synthesis. Muscles incubated with myostatin exhibited an increase in proteolysis with an increase of *Atrogin-1, MuRF1* and *LC3* genes. Extensor digitorum longus muscles and C2C12 myotubes exhibited a reduction in protein turnover. Cardiomyocytes showed an increase in proteolysis by activating autophagy and the ubiquitin proteasome system, and a decrease in protein synthesis by decreasing P70S6K. The effect of myostatin on protein metabolism is related to fiber type composition, which may be associated to the extent of atrophy mediated effect of myostatin on muscle.

## Introduction

Myostatin, also known as GDF-8, belongs to the TGF-β superfamily of ligands, which comprises at least 30 genes in mammals. It was first described in 1997 by McPherron et al. (1) as a novel specific autocrine/endocrine negative regulator of skeletal muscle mass, since its embryonary deletion promoted a large increase in muscle mass as a result of hyperplasia and hypertrophy. Later, myostatin was also identified to be expressed in fetal and adult murine heart in a lesser extent, but its expression can be upregulated under pathological conditions like myocardial infarction (1,2).

It is known that myostatin can interfere with protein synthesis as well as protein breakdown in proliferating and adult myofibers (3,4). However, there are conflicting results in the literature concerning the ability of myostatin to trigger protein degradation and/or impair protein synthesis (5,6).

The three main proteolytic processes involved in the control of muscle protein metabolism in mammals are the lysosomal, the Ca^2+^-dependent, and the ubiquitin-proteasome (UPS) systems. The acid hydrolases, named cathepsins, in lysosomes degrade the majority of extracellular and membrane proteins taken up by endocytosis as well as cytoplasmic proteins and organelles by autophagy (7,8). Two autophagic-related genes, the microtubule-associated protein light chain 3 (LC3) and the gamma-amino butyric acid receptor-associated protein (GABARAP), are indispensable for autophagic process since they anchor the lysosomal membrane to the autophagosome (9,10). On the other hand, the UPS degrades most intracellular proteins (11). It consists of well-controlled actions of enzymes that link chains of ubiquitin (UB) to the protein that will be degraded by proteasome 26S (12). MAFbx/atrogin-1 and MuRF1 are two E3 ligases involved in the ubiquitination of proteins, directing them to proteasome. Gomes et al. and Bodine et al. (13,14) have shown that these two E3 ligases are upregulated during several atrophy conditions.

In the present study, we compared protein turnover among different types of muscle fibers in the presence of myostatin. We used 2 muscles from rat: soleus (which has a greater proportion of type I fibers, oxidative) and extensor digitorum longus (EDL, which has a greater proportion of type II fibers, glycolytic). We also used C2C12 cells and primary culture of cardiomyocytes.

Here, we found that myostatin increased the phosphorylation of Smad3 in soleus and EDL muscles and promoted an increase in proteolysis in soleus and a decrease in protein turnover in EDL and C2C12 cell culture. These responses were associated with upregulation of Atrogin-1 in both muscles and C2C12 cells, and *LC3* gene only in soleus. In addition, we saw that myostatin activated proteolysis and inhibited protein synthesis in primary culture of neonatal cardiomyocytes and led to an increase in LC3-II and in the amount of polyubiquitinated proteins in parallel with a decrease in p-P70S6K.

## Material and Methods

### Animals and treatment

Because the incubation procedure required intact muscles of a sufficient thinness to allow for the adequate diffusion of metabolites and oxygen, 4-week-old male Wistar rats (∼80 g) were used in all experiments. Animals were housed in a room with 12:12 h light/dark cycle (starting at 6:00 am) at 25±2°C and were given free access to water and lab chow diet (NUVLAB, CR1; Nuvital, Brazil) for at least 2 days before the experiments. Rats were killed by cervical dislocation. All of the experiments and protocols were performed in accordance with the ethical principles for animal research adopted by Brazilian College of Animal Experimentation (COBEA) and were approved by Ethics Committee in Animal Research (CETEA; No. 063/2011) of the Faculdade de Medicina de Ribeirão Preto, Universidade de São Paulo, Brazil.

### Cell culture

Murine C2C12 muscle cells were grown in Dulbecco's modified Eagle's medium (DMEM) + 10% fetal bovine serum. Myoblasts were differentiated by shifting medium to DMEM containing 2% horse serum. Cells were maintained in differentiation media for 96 h. Myotube starvation was induced by Hanks balanced salt solution incubation plus glucose (4.5 g/L).

### Primary cultures of neonatal cardiac myocytes

Hearts of neonatal (1-day old) Wistar rats were excised, and the ventricles were minced and transferred to a sterile buffer. The tissue was then subjected to 6 to 7 subsequent enzymatic digestions with type IV collagenase, at 37°C for 12 min. The solution obtained from each digest was then transferred to a tube containing 1 mL of newborn calf serum (NCS) and centrifuged at 300 *g* for 5 min at 24^o^C. Each cell pellet was resuspended in NCS, and dissociated cells were pooled. To separate myocytes from non-myocytes, the cell suspension was layered onto discontinuous Percoll density gradients consisting of 2 phases. After washing to remove all traces of Percoll, the myocytes were cultured in DMEM containing 5% fetal calf serum, penicillin and streptomycin (P/S, 1%), and 10% horse serum for 48 h (15). The experiments were performed the next day. Myocytes were cultured in DMEM plus 10% of fetal bovine serum with or without myostatin for 24 h, since the lack of serum increases the mortality of these cells over such a long period.

### Protein breakdown in cultured cells

Cells were incubated with 0.05 μCi/mL L-[U-^14^C]-tyrosine for 36 h to label cellular proteins. The media were then switched to the chase media containing 2 mM of unlabeled tyrosine and incubated for 2 h. Myotubes or myocytes were then incubated with fresh chase media containing myostatin for 2, 4, 6 12, and 24 h. Aliquots (200 μL) of culture medium were taken at specific times for quantitation of L-[^14^C]-tyrosine release. Proteins were precipitated at 4°C with trichloroacetic acid (TCA, 10% final concentration) and centrifuged at 11,000 *g* for 5 min at room temperature (24^o^C). The precipitate was solubilized by lysis solution (1% Triton X-100 and 1 N NaOH). Radioactivity was measured in the TCA-soluble supernatant and in the proteins found in the pellet (TCA-insoluble fraction). Total radioactivity is the sum of the residual radioactivity in cell proteins and the TCA-soluble radioactivities at different time points. Protein breakdown is reported as the percentage of L-[^14^C]-tyrosine released at different times in relation to total L-[^14^C]-tyrosine incorporated at each time.

### Protein synthesis in cultured cells

L-[^14^C]-tyrosine was added in the medium (0.05 µCi/mL) in the last 2 h of all experiments. Cells were then washed with PBS and 10% TCA was added to the cells. They were collected and centrifuged at 11,000 *g* for 15 min at 4°C and the incorporated L-[^14^C]-tyrosine in protein was measured after solubilization with lysis buffer. The amount of protein labeled was corrected to the total of protein, measured by Bradford reagent.

### Incubation procedure of isolated muscles

The soleus and EDL muscles were rapidly dissected, avoiding any damage to the muscles. The muscles were maintained at resting length by pinching their tendons in aluminum or plastic supports. They were incubated at 37°C in Krebs-Ringer bicarbonate buffer, pH 7.4, equilibrated with 95% O_2_ and 5% CO_2_ containing glucose (5 mM) in the presence of cycloheximide (0.5 mM) to prevent protein synthesis and the reincorporation of tyrosine back into proteins. Tissues were pre-incubated for 1 h and then incubated for 2 h with or without myostatin in fresh medium of identical composition. After the 2 h, the muscles were incubated again in fresh and identical medium.

### Protein degradation in isolated muscles

The rate of overall proteolysis was determined by measuring the rate of tyrosine release in the incubation medium. Because muscles cannot synthesize or degrade tyrosine, its release reflects the rate of protein breakdown. Tyrosine was assayed by a fluorimetric method as previously described (16).

### Protein synthesis in isolated muscles

To measure rates of protein synthesis in soleus and EDL, L-[U-^14^C]-tyrosine (0.05 µCi/mL) was added in Krebs-Ringer bicarbonate buffer without cycloheximide only after the pre-incubation. Muscles were incubated for 2 h at 37^o^C with or without myostatin, the medium was changed to an identical medium and incubated for an additional 2 h. The muscles were then used to measure the specific activity of acid-soluble tyrosine (intracellular tyrosine pool) by measuring the radioactivity and the concentration of tyrosine by a previously described method (16). After measurement of the radioactivity incorporated into protein of the same muscle, the rate of synthesis was calculated using the specific activity of the intracellular pool of tyrosine, assuming that there was no recycling of the label during the incubation period (17,18).

### Western blotting analysis

Soleus and EDL muscles were immediately frozen in liquid nitrogen after collection. Muscles were homogenized in 50 mM Tris–HCl buffer, pH 7.4, at 4°C containing 150 mM NaCl, 1 mM EDTA, 1% Triton X-100, 1% sodium deoxycholate, 1% SDS, 10 mM sodium pyrophosphate, 100 mM sodium fluoride, 10 mM sodium orthovanadate, 5 μg/mL of aprotinin, 1 μg/mL of leupeptin and 1 mM phenylmethylsulfonyl fluoride. The homogenate was centrifuged at 14,000 *g* for 20 min at 4°C, retaining the supernatant, and protein content was determined using BSA as a standard (19). An equal volume of sample buffer (20% glycerol, 125 mM Tris-HCl, 4% SDS, 100 mM dithiothreitol, 0.02% bromophenol blue, pH 6.8) was added to the supernatant and the mixture was boiled. Fifty micrograms of proteins were subjected to SDS-PAGE analysis on 8, 10, 14, or 18% acrylamide gels depending on the protein. Control and experimental samples were always run on the same gel. Gels were electroblotted onto nitrocellulose membranes (20) and blotted with different primary antibodies. Primary antibody was detected by peroxidase-conjugated secondary antibody and visualized by ECL reagents and detected by Molecular Imager ChemiDoc XRS+ (BioRad, Brazil). Band intensities were quantified using the Image Lab software (BioRad).

### Quantitative polymerase chain reaction

RNA was isolated from soleus and EDL muscles using TRIzol (Invitrogen, USA). Reverse transcription into cDNA was performed using 2 µg of total cellular RNA, 20 pmol oligo(dT) primer (Invitrogen), and reverse transcriptase (Advantage ImProm-II; Promega, USA). Real-time polymerase chain reaction (PCR) was carried out on a sequence detection system (Model ABI-7000; Applied Biosystems, USA), using the SuperScript III Platinum SYBR Green One-Step RTqPCR Kit with ROX (Invitrogen) with the primer of interest. Transcript levels of target gene were normalized with *gapdh*. Relative fold change in expression was calculated using the delta delta cycle threshold (ΔΔCT) method.

### Statistical analyses

Data were analyzed using one-way ANOVA for dose-response and among different treatments in cells. Student's *t*-tests were performed for comparison between groups. Two-way ANOVA was used to analyze the time-dependent proteolysis in C2C12 and cardiomyocytes. Data are reported as means±SE. Differences were considered statistically significant at P*<*0.05.

## Results

### Dose-response of myostatin incubation on proteolysis of isolated soleus, EDL and in C2C12 cells

We show here the results concerning the early time point that myostatin triggers a response in proteolysis.

**Figure 1.**
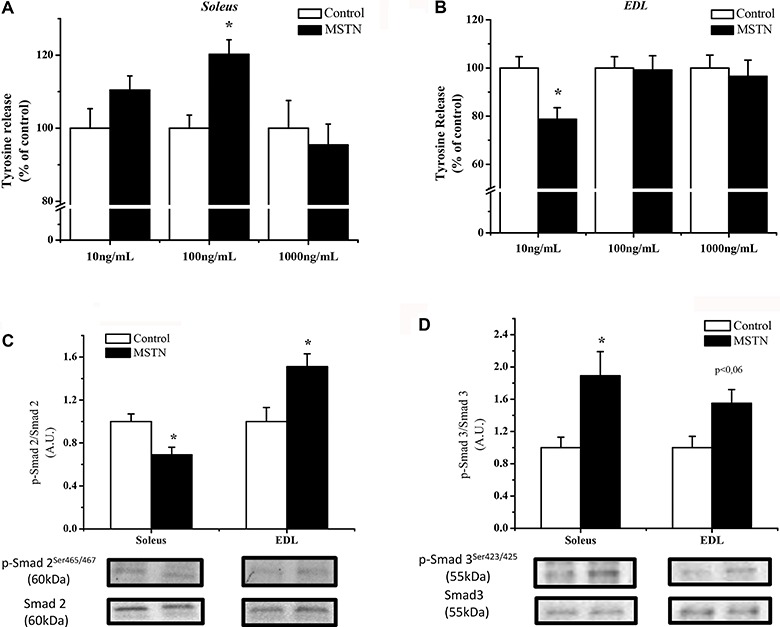
Overall proteolysis in (*A*) soleus and (*B*) extensor digitorum longus (EDL) muscles from rats incubated with myostatin (MSTN) for 4 h at different concentrations. Data are reported as means±SE (n=7). *P<0.05 MSTN *vs* Control, which is considered 100% (*t*-test). The control values of released tyrosine are (nmol·mg^−1^·2h^−1^): 0.302±0.016, 0.514±0.020, and 0.465±0.035 for soleus, and 0.268±0.012, 0.439±0.021, 0.370±0.020 for EDL at doses of 10, 100, and 1000 ng/mL, respectively. Phosphorylation status of Smad2 (*C*) and Smad3 (*D*) from soleus (100 ng/mL) and EDL (10 ng/mL) incubated with MSTN. A.U.: arbitrary units. *P<0.05 MSTN *vs* Control (*t*-test).

In the presence of 100 ng/mL of myostatin, soleus muscles showed a 20% increase in overall proteolysis after 4 h of incubation (nmol of tyrosine·mg^−1^·2 h^−1^; 0.617±0.021 *vs* 0.514±0.020 in controls; Figure 1A). Surprisingly, the incubation of EDL with 10 ng/mL of myostatin led to a decrease by 21% in overall proteolysis (0.211±0.013 *vs* 0.268±0.012 in controls; Figure 1B) after 4 h of incubation.

To make sure that our myostatin was triggering intracellular signaling, we evaluated the phosphorylation levels of Smad2 and 3 after 4 h of incubation with myostatin. Soleus muscles incubated with 100 ng/mL of myostatin for 4 h exhibited an increase in p-Smad3 by 89% and a 30% decrease in p-Smad2. EDL muscles incubated with 10 ng/mL of myostatin for 4 h showed an increase in both p-Smad3 and p-Smad2 by 50% (Figure 1C and D).

**Figure 2.**
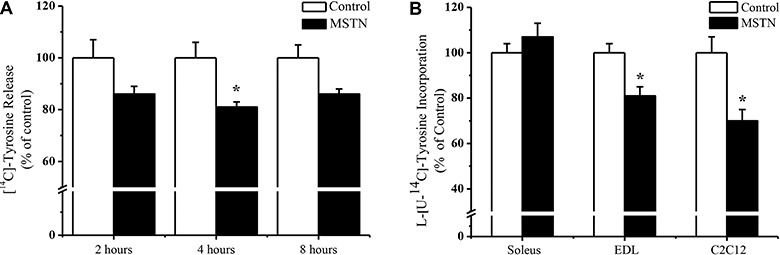
*A*, Overall proteolysis in C2C12 cells incubated with myostatin (MSTN) (100 ng/mL) after 2, 4, and 8 h. Data are reported as means±SE of labeled tyrosine released into the medium related to total amount of labeled tyrosine incorporated into proteins (%). Control was considered to be 100%, n=5. *P<0.05 MSTN *vs* Control (DMEM). *B*, Rate of protein synthesis in soleus and EDL muscles, and C2C12 cells incubated with MSTN at 100, 10, and 100 ng/mL, respectively, for 4 h. Data are reported as the means±SE of percentage of tyrosine incorporated into total protein (n=7). Control was considered to be 100%. *P<0.05 MSTN *vs* Control, *t*-test.

As seen in Figure 2A, myotubes incubated in the presence of myostatin (100 ng/mL) exhibited a decrease in 20% in overall proteolysis after 4 h.

### Rate of protein synthesis in skeletal muscles and C2C12 cells incubated with myostatin

After 4 h of incubation with myostatin, no difference was detected in soleus muscle and a ∼20% decrease in protein synthesis was seen in EDL muscle and C2C12 cells (Figure 2B).

### Gene expression in muscles and C2C12 cells incubated in the presence of myostatin

**Figure 3.**
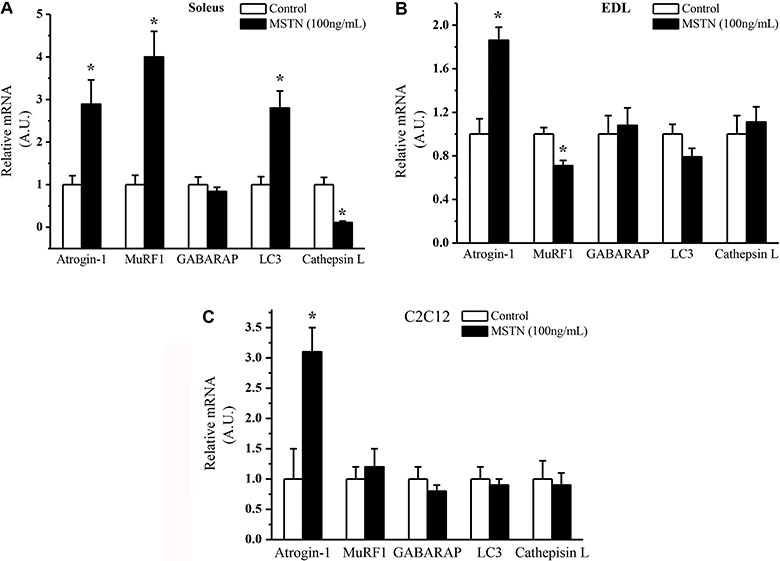
Gene expression of atrogin-1, MuRF1, GABARAP, LC3 and cathepsin L in *A*, soleus, *B*, extensor digitorum longus (EDL), and *C*, C2C12 cells incubated with myostatin (MSTN) for 4 h. Data are reported as the means±SE of percentages (n=6). Controls were considered to be 100%. *P<0.05 MSTN vs Control (*t*-test). A.U.: arbitrary units.

To link the different effects observed in protein turnover with the expression of genes related to UPS and autophagy proteolytic systems, an analysis of atrogenes and autophagic genes was performed. Soleus muscles incubated with 100 ng/mL for 4 h exhibited a 3-fold increase in atrogin-1, MuRF-1, and LC3 and a remarkable decrease in cathepsin L (Figure 3A). On the contrary, EDL incubated with 10 ng/mL of myostatin showed an increase only in atrogin-1 and a modest decrease in MuRF-1 with no changes in LC3, GABARAP and cathepsin L (Figure 3B). In C2C12 cells, myostatin incubation at 100 ng/mL only increased atrogin-1 expression 3-fold with no changes in MuRF-1, LC3, and GABARAP (Figure 3C).

### Overall proteolysis and rate of protein synthesis in primary cultures of neonatal rat cardiomyocytes incubated with myostatin

**Figure 4.**
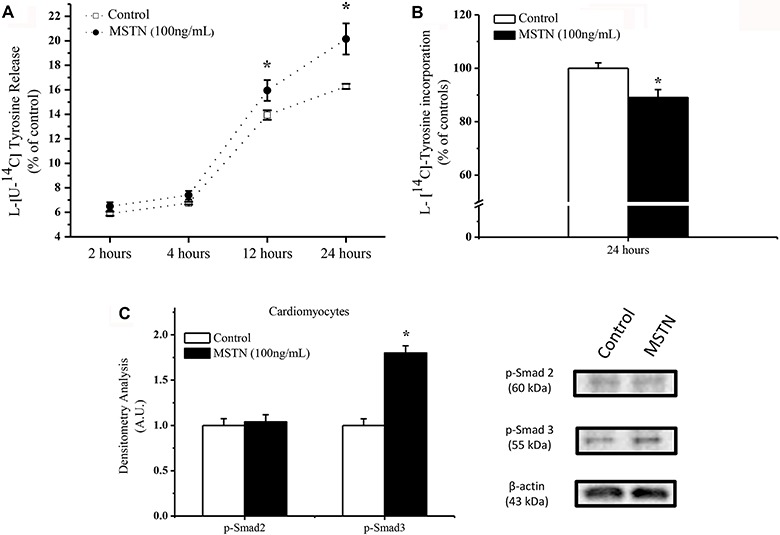
*A*, Overall proteolysis over time in cardiomyocytes incubated with myostatin (MSTN, 100 ng/mL). Data are reported as means±SE of the percentage of labeled tyrosine related to the total amount of labeled tyrosine incorporated into proteins. Control was considered to be 100%, n=6. *B*, Rate of protein synthesis in cardiomyocytes after 24 h of incubation with myostatin (100 ng/mL). Data are reported as means±SE of the percentage of tyrosine incorporated into total protein (n=7). Control was considered to be 100%. *C*, Phosphorylation status and densitometry analysis of Smad2 and Smad3 in cardiomyocytes incubated with myostatin for 4 h. Densitometry was corrected to β-actin. *P<0.05 MSTN *vs* Control (*t*-test).

Since the effects of myostatin in heart are lesser known than in skeletal muscle, a time-dependent proteolysis was performed to seek at which point myostatin would trigger a response in protein turnover. Different from what happened in skeletal muscle, short-time incubation with myostatin (4 h) of cardiomyocytes primary culture did not affect overall proteolysis (Figure 4A) nor protein synthesis (data not shown). However, cardiomyocytes incubated in the presence of myostatin exhibited an increase in protein degradation after 12 and 24 h (Figure 4A). The rate of protein synthesis was measured after 24 h and we could see a decrease by ∼20% in tyrosine incorporation in cardiomyocytes incubated with 100 ng/mL of myostatin (Figure 4B). The 24 h incubation with myostatin increased the phosphorylation status of p-Smad3 by 2-fold with no changes in p-Smad2 (Figure 4C).

### Protein content of poly-Ub proteins, LC3 and GABARAP

Since we observed an increase in proteolysis and a decrease in protein synthesis in cardiomyocytes, we evaluated some key parameters of both processes. The direct effect of myostatin on protein degradation and synthesis is less understood when compared to skeletal muscles. Therefore, in this section we measured some components that can help us to explain the responses seen in protein turnover.

**Figure 5.**
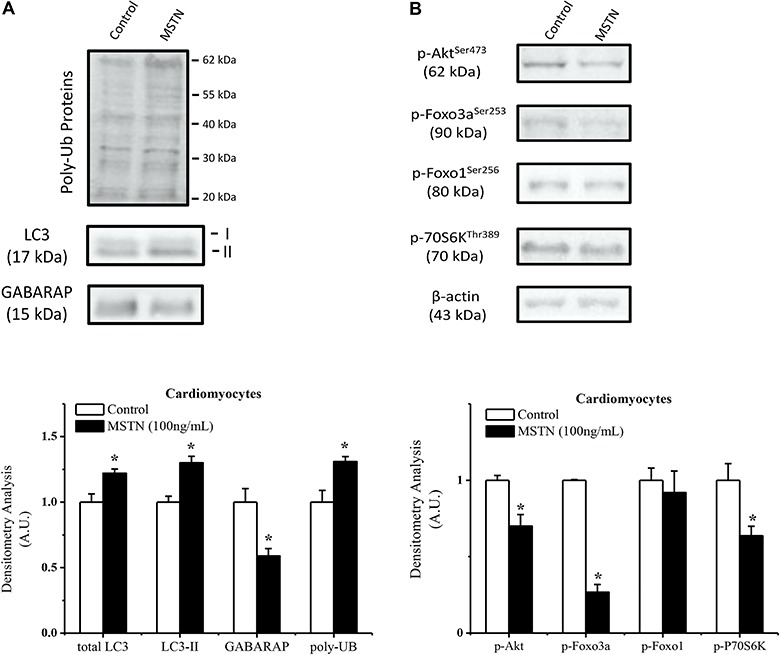
*A*, Protein content of ubiquitinated (UB) proteins, total LC3 and form II, and GABARAP from cardiomyocytes incubated with myostatin (MSTN) (100 ng/mL) for 24 h with densitometry analysis of these proteins corrected to β-actin. Control was considered to be 1. Data are reported as means±SEM (n=5). *B*, Phosphorylation status of Akt, FoxO3a, FoxO1, P70S6K in cardiomyocytes incubated with myostatin for 24 h with densitometry analysis of these proteins corrected to their respective total proteins. Control was considered to be 1. Data are reported as means±SE (n=5). *P<0.05, *t*-test.

Cardiomyocytes incubated with 100 ng/mL of myostatin for 24 h exhibited a 30% increase in the amount of proteins target with a polyubiquitin chain and an increase in the LC3 total protein and LC3-II by 20 and 30%, respectively (Figure 5A). Conversely, GABARAP protein content was reduced by 40% in cardiomyocytes incubated with myostatin for 24 h (Figure 5A).

### Phosphorylation status of Akt, FoxO and P70S6K

It is known that myostatin can inhibit Akt phosphorylation and induce FoxO transcriptional activity in skeletal muscle (21) and that myostatin is able to decrease protein synthesis via P70S6K (5) in C2C12 cells. Based on this, we measured the levels of Akt, FoxO1 and P70S6K phosphorylation (Figure 5B).

The early onset trigger in Smad phosphorylation promoted a reduction in p-Akt (30%), and a remarkable decrease in p-FoxO3a (70%) and p-P70S6K (40%) after 24 h of incubation with myostatin (Figure 5B).

## Discussion

It is well established that myostatin is a potent negative regulator of skeletal muscle mass. Since its discovery, different studies have shown the mechanism by which myostatin promotes the loss of proteins in skeletal muscles. Despite this very clear phenomenon, there is some conflicting evidence concerning which processes are altered in the presence of myostatin. The differences may be due to different concentrations of myostatin used, myostatin isoform (active homodimer or full-length) and methodologies.

Myostatin activity is regulated at several levels. First, myostatin expression is limited to a few cell types, including skeletal muscle, heart and adipose tissues (1,2). Second, myostatin is synthesized as a precursor protein that remains inactive until it is modified by several post-translational events (22). Third, multiple extracellular factors can modulate the access and activity of myostatin to cell surface receptors (23).

In the present study, we have used a full-length *Escherichia coli*-derived myostatin as confirmed by Ponceau staining (Supplementary Figure 1) and the protein turnover was evaluated in isolated muscles from young rats, C2C12 and primary culture of neonatal cardiomyocytes. To our knowledge, this is the first time that different types of muscle fiber are compared in terms of protein turnover when incubated with myostatin.

Myostatin is a well-known negative regulator of skeletal muscle mass due to its effects in protein synthesis as well proteolysis. The literature shows some conflicting results concerning activation of proteolysis and inhibition of synthesis. For instance, Lokireddy et al. (6) showed that human recombinant myostatin when incubated with human myoblast cells for 24 h promoted an increase in both atrogin-1 and MuRF-1 expression and this was associated with an increase in protein degradation. Herein, we reported that soleus when incubated with myostatin for 4 h exhibited an increase in both atrogin-1 and MuRF-1, which was also associated with an increase in protein degradation. We suggest that only protein degradation was activated upon myostatin treatment in an oxidative muscle such as soleus, which may reflect decreased action of myostatin on this type of muscle fiber.

Lee et al. (24) demonstrated that myostatin promotes autophagy in C2C12 cells in the absence of horse serum in the medium. They have also shown an increase in LC3 and autophagosome number in those cells incubated with myostatin. In the present study, it was possible to detect an increase in LC3 expression in soleus muscles in the presence of myostatin, which may indicate that myostatin can activate the autophagic process at least in oxidative muscles.

In contrast to soleus, EDL exhibited a different response upon myostatin incubation. A reduction in overall proteolysis was accompanied by a decrease in protein synthesis. Similar results in overall proteolysis in C2C12 cells were obtained, i.e., a decrease in protein turnover. This result agrees with previous studies from the literature in which glycolytic fibers are more sensitive to myostatin actions than oxidative type I fibers (25). In addition, myostatin promotes glycolysis by increasing expression of several genes involved in regulating glucose metabolism (26). These effects may help explain the differences in protein turnover between soleus and EDL muscle in our study.

Different from Lokireddy et al. (6), Trendelenburg et al. (5) found a decrease in the expression of atrogin-1 and MuRF-1 in skeletal myotubes when incubated with myostatin, along with a decrease expression of MyoD and myogenin, two genes involved in the differentiation of myoblasts. The authors postulated that myostatin was able to block muscle differentiation program even when incubated in already mature myotubes. They attributed these effects to the decrease Akt phosphorylation status, which could inhibit the rate of protein synthesis. These findings could help us explain the decrease in protein turnover in EDL muscle and C2C12 cells in this study. We may suggest that myostatin triggered responses that caused the muscle to be in a quiescent status as reported by Joulia et al. (27), who have shown that myostatin does reduce cell growth and differentiation.

In addition, Lokireddy et al. (28) showed that myostatin treatment of C2C12 cells was able to induce 47 atrogin-1 target proteins and among those they found a cluster of protein synthesis components that are targeted by atrogin-1 upon myostatin treatment. This may indicate that myostatin decreases protein synthesis through the upregulation of atrogin-1, as observed in our study when EDL and C2C12 cells exhibited a decrease in protein synthesis with an increase in atrogin-1 expression. On the other hand, other authors (29,30) have shown that MuRF1 was associated with the ubiquitination and degradation of sarcomeric proteins, leading to a remarkable increase in proteolysis. In our study, we could not detect an increase in MuRF1 in C2C12 cells and a decrease in the expression was seen in EDL muscle when incubated with myostatin. Only soleus had MuRF1 upregulated upon myostatin treatment and this fact was directly associated with an increase in protein degradation. Taken together, these findings support the idea that atrogin-1 is more involved in targeting and ubiquitinating proteins involved in protein synthesis and MuRF1 would be more responsible for sarcomeric degradation of proteins, which are the major class of proteins in skeletal muscle.

Cardiomyocytes exhibited an increase in protein breakdown and a decrease in protein synthesis only after 24 h of incubation with myostatin. In agreement with this latter finding, McKoy et al. (31) have found that protein synthesis from embryonic cardiomyocytes (E18) was decreased when incubated for 24 h with myostatin. Of note, these authors observed that the maximal activation of Smad2 in these cells occurred after 30 min and no changes were seen after 24 h. Herein, we detected an increase in p-Smad3 after 4 h but not after 24 h. Therefore, a feedback loop may take place in order to extinguish the signal at the level of the Smad phosphorylation (32), but the effects on protein synthesis are still seen later. On the other hand, Morissette et al. (33) did not find any difference in protein synthesis when the full-length form of myostatin were overexpressed in neonatal cardiomyocytes. However, these authors reported a suppression of protein synthesis driven by myostatin after phenylephrine (PE) stimulation. They attributed this fact to a myostatin-mediated inhibition of Akt, which is increased during PE treatment. In the present study, a reduction in protein synthesis in neonatal cardiomyocytes incubated with myostatin for 24 h was observed. In agreement with this, a decrease in P70S6K phosphorylation, a downstream target of Akt involved in protein synthesis, has been shown to be involved in the rapid hypertrophic growth through the control of myosin gene (34).

The finding that myostatin can increase proteolysis in cardiomyocytes is noteworthy since there is a lack of studies aiming this process in this muscle type. The effects of myostatin in heart have been attributed so far to an inhibition of protein synthesis in cases of pathological hypertrophy, such as heart failure (35). Therefore, it has been postulated that myostatin acts to counteract the pathological hypertrophic effects as seen in stretch-induced IGF-1 secretion where myostatin expression is upregulated (36,37).

Following this line of evidence, it is feasible to postulate that myostatin can also increase proteolysis in order to alleviate any stressful stimuli to the heart. Herein, a decrease in FoxO3a phosphorylation was seen. This increase in proteolysis may be associated with an increase in the activity of autophagy and ubiquitin-proteasome systems, since an increase in LC3-II, which is the bound form to autophagosome membrane, and an increase in total polyubiquitinated proteins were observed in cardiomyocytes incubated with myostatin for 24 h. More studies should be conducted in order to consider the above idea because the model used in this study did not take into account any model of pathological hypertrophy.

Taken together, the present study showed that incubation of isolated muscles from rats, C2C12 cells and cardiomyocytes with myostatin led to a different response pattern in protein turnover. First, soleus (oxidative fiber) exhibited an increase in proteolysis associated with an increase in atrogin-1, MuRF-1 and LC3. Second, EDL exhibited a decrease in protein turnover with an increase only in atrogin-1 expression, results that were similar in C2C12 cells. In addition, cardiomyocytes incubated with myostatin for 24 h exhibited a decrease in synthetic rate and an increase in proteolysis. The decrease in phosphorylation levels of Akt, P70S6K and FoxO3a and the increase in LC3-II and poly-Ub proteins may indicate that UPS and autophagy are increased in this cell line.

## Supplementary material

Click here to view [pdf].
